# Supplementary material for: Structural insights into SSNA1 self-assembly and its microtubule binding for centriole maintenance
Source: Nat Commun. 2025 Aug 13;16:7512. doi: 10.1038/s41467-025-62696-9 (PMC12350680; doi:10.1038/s41467-025-62696-9)
Supplement: Supplementary file 1 — Supplementary Information [file 41467_2025_62696_MOESM1_ESM.pdf]

Structural insights into SSNA1 self-assembly and its microtubule binding for centriole maintenance

Lorenzo Agostini[1*], Jason A. Pfister[2*], Nirakar Basnet[1#], Jienyu Ding[1], Rui Zhang[3], Christian Biertümpfel[1], Kevin F. O'Connell[2**], Naoko Mizuno[1,4**]**

[1] Laboratory of Structural Cell Biology, National Heart, Lung, and Blood Institute, National Institutes of Health, 50 South Dr., Bethesda, MD, 20892, USA

[2] Laboratory of Biochemistry and Genetics, National Institute of Diabetes and Digestive and Kidney Diseases, National Institutes of Health, 8 Center Dr., Bethesda, MD, 20892, USA

[3]Department of Biochemistry and Molecular Biophysics, Washington University in St. Louis, School of Medicine, St. Louis, MO, USA

[5] National Institute of Arthritis and Musculoskeletal and Skin Diseases, National Institutes of Health, 50 South Dr., Bethesda, MD, 20892, USA

* equal contribution

** correspondence
Christian Biertuempfel: christian.biertuempfel@nih.gov
Kevin F O'Connell kevino@intra.niddk.nih.gov
Naoko Mizuno: naoko.mizuno@nih.gov

# Current Address: Institute for Protein Innovation, Boston, MA, 02115, USA

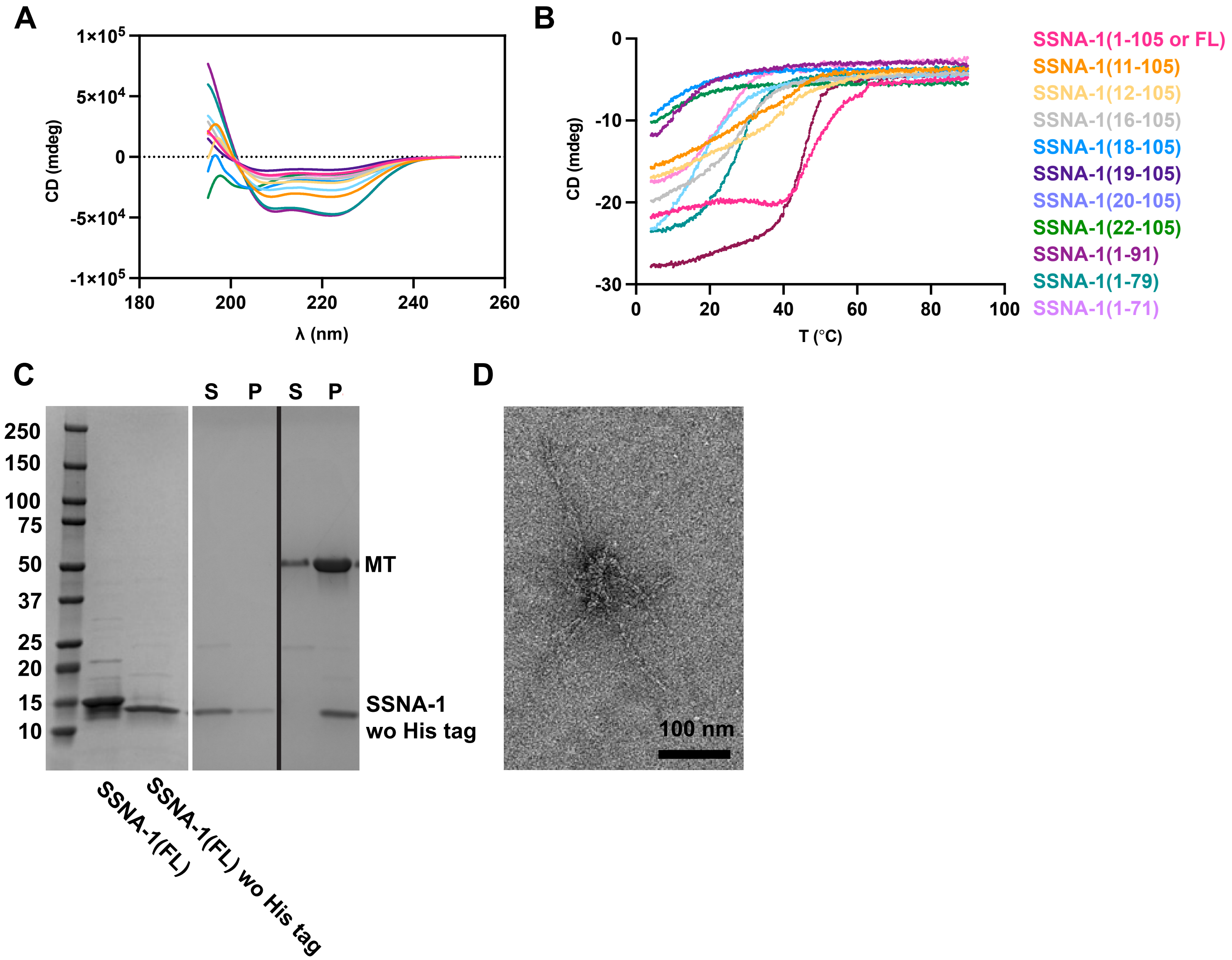

Supplementary Figure 1. Biophysical and biochemical characterization of SSNA-1 and binding of the microtubules independent of His tag. **A.** Circular dichroism (CD) spectra of various SSNA-1 constructs were recorded as the average of four scans and are shown as mean residue ellipticity [θR] (deg·cm²·dmol⁻ ¹). Colors correspond to the respective constructs shown in panel **B**. Thermal unfolding of SSNA-1 variants monitored by CD, showing melting profiles as a function of temperature. **C.** SDS-PAGE analysis confirms 3C protease cleavage of the His tag from SSNA-1. A microtubule co-sedimentation assay shows that tag-free SSNA-1 still binds to microtubules, indicating His-tag independence. **D.** Negative-stain electron microscopy showing tag-free SSNA-1 forms fibrils, which accounts for its sedimentation in the absence of microtubules.

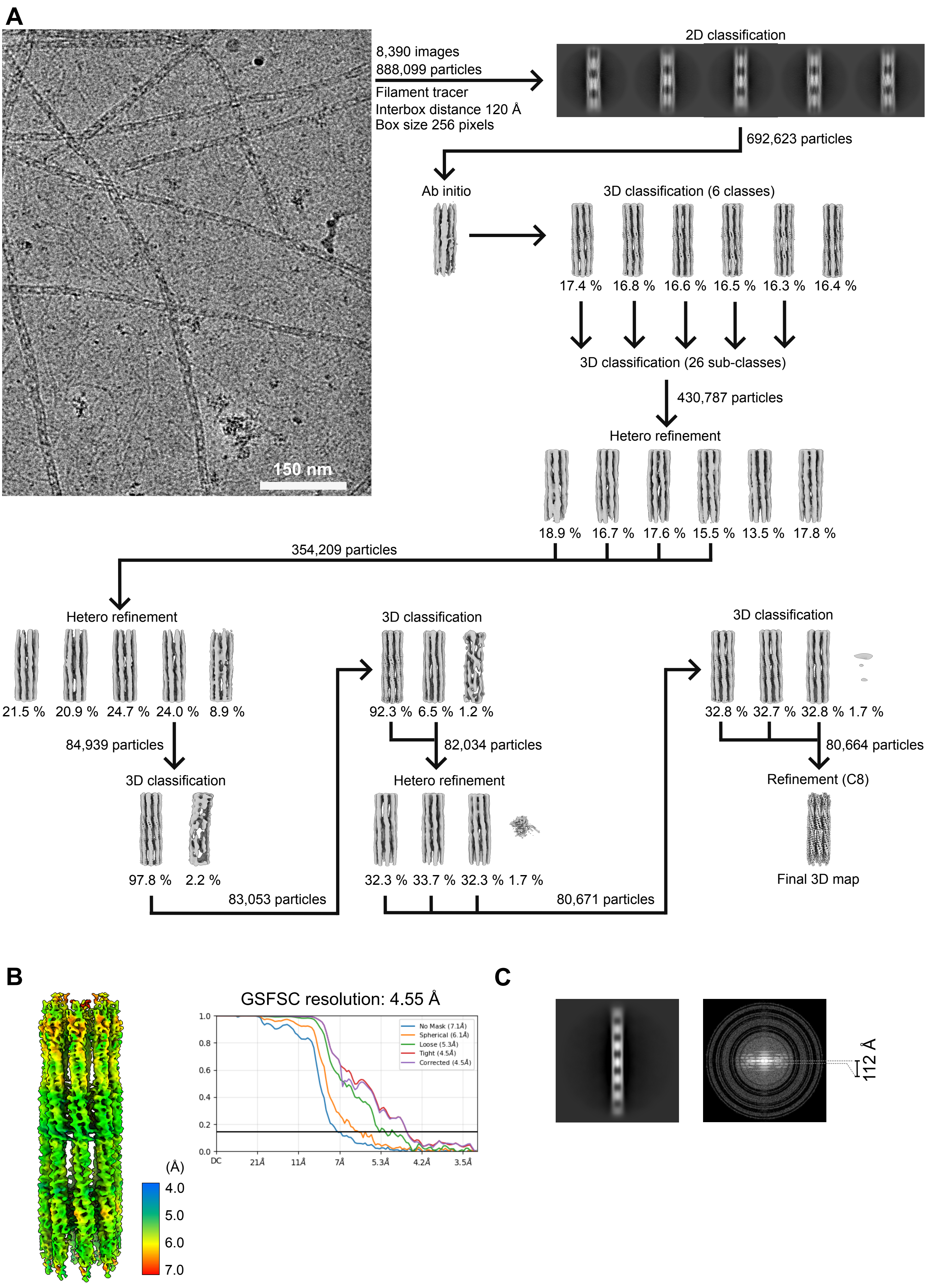

Supplementary Figure 2. 3D reconstruction of SSNA-1(3E). **A.** Cryo-EM data analysis workflow. **B.** Local resolution map of SSNA-1(3E) colored from blue (4 Å), green (5 Å), yellow (6) to red (7 Å) with an overall resolution of 4.55 Å at a gold-standard FSC of 0.143. **C.** Left: 2D class average of SSNA-1(3E) with a box size of 512 pixel (1.648 Å/pixel). Right: Averaged power spectrum of SSNA-1(3E) with layer lines in grey showing a helical periodicity of 112 Å.

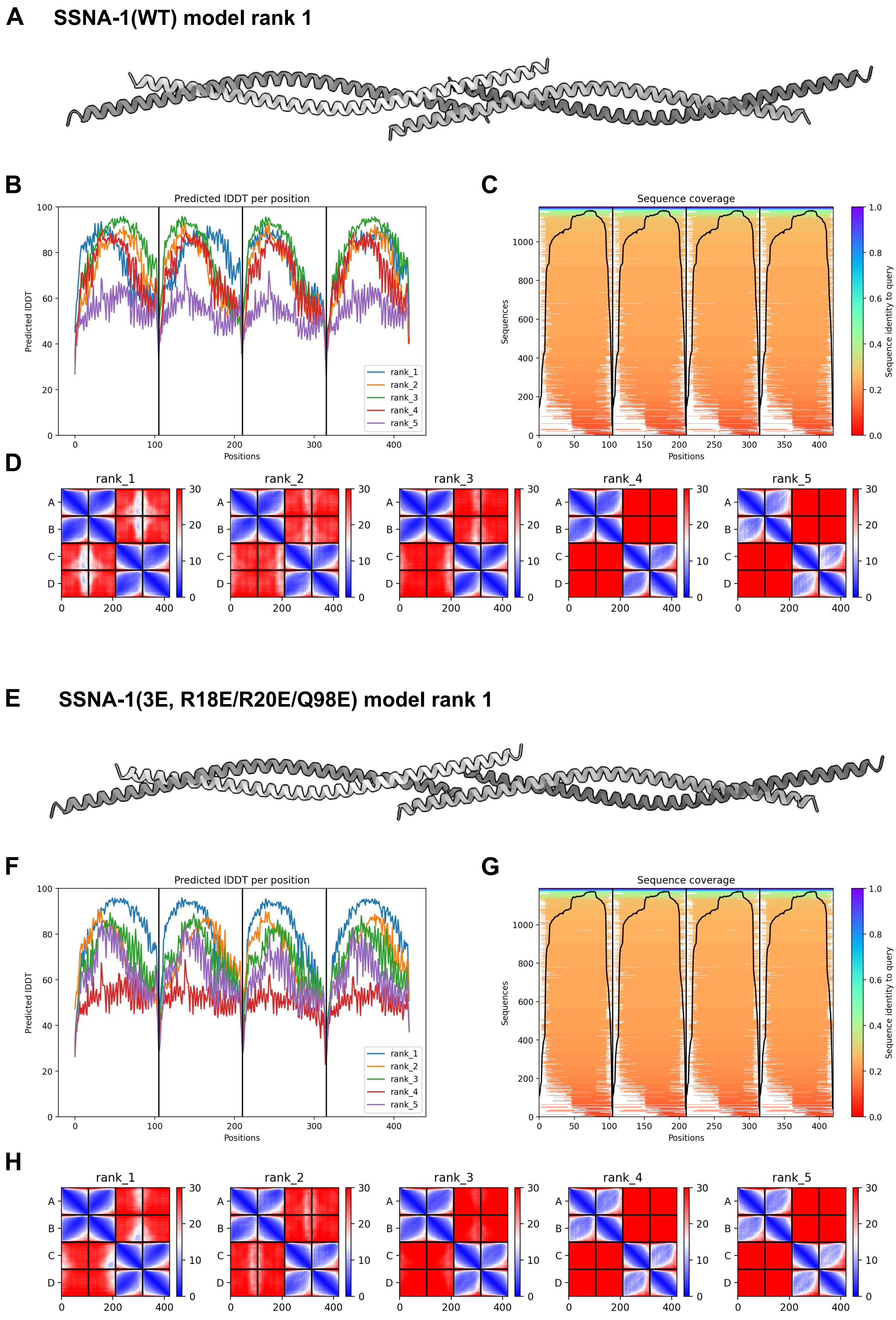

Supplementary Figure 3. AlphaFold prediction of SSNA-1(WT) and SSNA-1 (3E). **A.** Rank 1 model for SSNA-1 (WT). **B.** Predicted local distance difference test (pLDDT) for the top 5 ranked models of SSNA-1 (WT). **C.** Multiple sequence alignment (MSA) for SSNA-1 (WT) summarized as heatmap. **D.** Predicted aligned error (PAE) for the top 5 ranked models of SSNA-1 (WT). **E.** Rank 1 model for SSNA-1 (3E). **F.** Predicted local distance difference test (pLDDT) for the top 5 ranked models of SSNA1 (3E). **G.** Multiple sequence alignment (MSA) for SSNA1 (3E) summarized as heatmap. **H.** Predicted aligned error (PAE) for the top 5 ranked models of SSNA1 (3E).

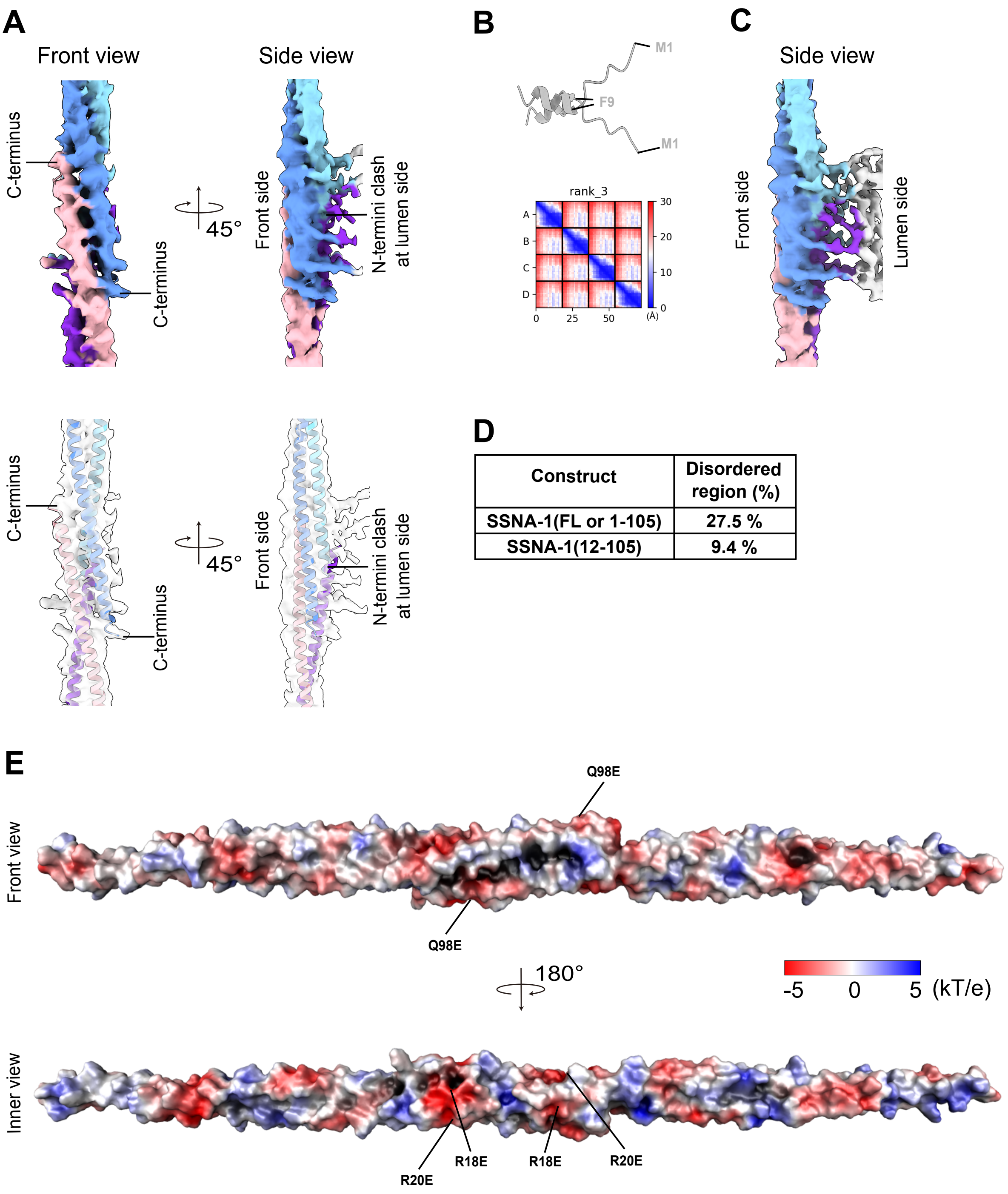

Construct	Disordered region (%)
SSNA-1(FL or 1-105)	27.5 %
SSNA-1(12-105)	9.4 %

Supplementary Figure 4. Structural analysis and modelling of SSNA-1(3E). **A.** Two views of the 3D reconstruction of SSNA-1(3E) fibril. Top: The cryo-EM map is colored according to the position of helical strands of the structural model. Bottom: The cryo-EM map is half transparent to show the structural model. Residues M1-S8 are predicted to be part of the coiled-coil by AlphaFold but they would clash when fit into the EM density. The cryo-EM map corresponding to this location shows an extra density from residue F9 protruding to the inner lumen and connecting to the disordered luminal region. **B.** Top: AlphaFold prediction of SSNA-1(1-18) folding into the inner lumen of the filament (disordered part in the structure). Bottom: Plot of the predicted alignment error (PAE) of the AlphaFold prediction of SSNA-1(1-18). **C.** Side view of the SSNA-1(3E) cryo-EM map where a single fibril connects to the inner lumen density formed by the N-termini. **D.** Proportion of non-helical (i.e. disordered) regions as measured by the α-helical contribution in circular dichroism (CD) for SSNA-1(FL) and SSNA-1(12-105). **E.** Electrostatic surface potential map of the structural model of SSNA-1(3E) residues 7-105 calculated with APBS and PDB2PQR56 in PYMOL (Schroedinger). The highlighted residues (R18E/R20E/Q98E) were chosen as point mutations to create the structurally amenable form SSNA-1(3E).

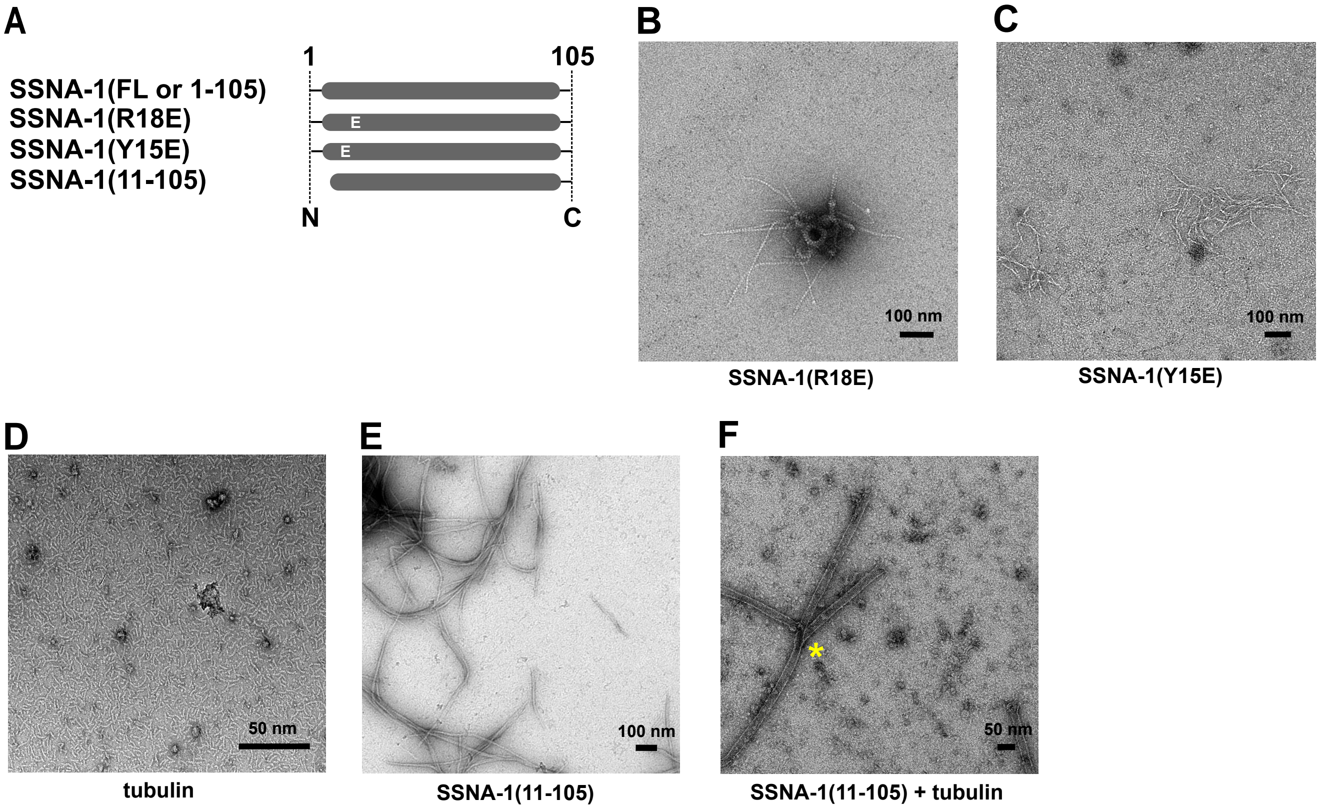

Supplementary Figure 5. Additional biophysical and biochemical characterization of SSNA-1. **A.** Schematic of additional SSNA-1 constructs generated to assess functional regions involved in the self-assembly process. **B.** Representative negative-staining EM images of SSNA-1(R18E). **C.** Representative negative-staining EM image of SSNA-1(Y15E). **D.** Representative negative-staining EM image of tubulin (using the conditions of the microtubule-branching assay) showing that microtubules could not be polymerized in the absence of SSNA-1. E. Representative negative-staining EM image of SSNA-1(11-105). **F.** Representative negative-staining EM image of SSNA-1(11-105) mixed with tubulin under microtubule-branching conditions. The yellow asterisk indicates a microtubule branch.

Supplementary Table 1: Data collection and structure refinement statistics

**Data Set**	**SSNA-1(R18E/R20E/Q98E)**<br>**EMD-47147**<br>**PDB 9dsm**
**Data collection statistics**	
Microscope	ThermoFisher Scientific Krios G4
Energy filter	Gatan BioContinuum
Detector	Gatan K3 Summit
Grid type	Quantifoil Cu 200 R2/1
Magnification	105,000
Voltage (kV)	300
Electron dose ($e^-/Å^2$)	40.84
Dose rate (e^-/s/pixel)	14.862
Defocus Range (µm)	-0.5 to -2.3
Number of movie frames	40
Pixel size (Å)	0.412
Number of micrographs	10396
Number of particles (total)	888,099
Number of particles (final map)	80,664
FSC threshold	0.143
Global resolution (Å)	4.55
Local resolution (Å)	3.64 – 36.5
**Refinement statistics**	
Particles used for final map	80,664
Map sharpening B factor ($Å^2$)	178.83
Number of chains	32
Number of Residues	
Proteins	3168
Number of Atoms	
Proteins	26528
B-factors	
Protein	261.2
R.m.s deviations	
Bond lengths (Å)	0.002
Bond angles (˚)	0.433
Validation	
MolProbity score	1.63
Clashscore	13.27
Rotamer outliers (%)	0.00
CaBLAM outliers (%)	0.00
Cβ outliers (%)	0.00
CC(mask)	0.61
Ramachandran Plot	
Favored (%)	99.7
Allowed (%)	0.3
Outliers (%)	0.0

Supplementary Table 2. *C. elegans* strains used in this study.

N2	Wild type
OC908	*bsSi30 [pCW9: unc-119(+) pcdk-11.2::sfgfp::his-58::cdk-11.2 3' utr] II; bsIs20 [pNP99: unc-119(+) tbb-1p::mCherry::tbb-2::tbb-2 3'-utr]; bsIs2 [pCK5.5: Ppie-1::gfp::spd-2]*
OC1013	*bsSi30 [pCW9: unc-119(+) pcdk-11.2::sfgfp::his-58::cdk-11.2 3' utr] II; bsIs20 [pNP99: unc-119(+) tbb-1p::mCherry::tbb-2::tbb-2 3'-utr]; bsIs2 [pCK5.5: Ppie-1::gfp::spd-2]; ssna-1(bs182) / dpy-9(tm9713) kvs-5(tmIs1245)] IV*
OC1021	*zyg-1(bs197[zyg-1::spot] II*
OC1050	*ssna-1(bs218[ssna-1::C-tag]) IV*
OC1051	*zyg-1(bs197[zyg-1::spot] II; ssna-1(bs218[ssna-1::C-tag]) IV*
OC1138	*ssna-1(bs182)/ears-2(ve631[LoxP + myo-2p::GFP::unc-54 3' UTR + rps-27p::neoR::unc-54 3' UTR + LoxP]) IV*
OC1201	*ssna-1(bs246[Q98E]) IV*
OC1267	*ssna-1(bs284[Y15E]) IV*
OC1274	*ssna-1(bs286[R18E]) IV*
OC1276	*ssna-1(bs312[Y15E, Y97E])/ears-2(ve631[LoxP + myo-2p::GFP::unc-54 3' UTR + rps-27p::neoR::unc-54 3' UTR + LoxP]) IV*
OC1278	*ssna-1(bs314[R18E, Y97E])/ears-2(ve631[LoxP + myo-2p::GFP::unc-54 3' UTR + rps-27p::neoR::unc-54 3' UTR + LoxP]) IV*
OC1336	*ssna-1(bs355[Y97E])/ears-2(ve631[LoxP + myo-2p::GFP::unc-54 3' UTR + rps-27p::neoR::unc-54 3' UTR + LoxP]) IV*
OC1388	*ssna-1(bs381[Y15E, R18E])/ears-2(ve631[LoxP + myo-2p::GFP::unc-54 3' UTR + rps-27p::neoR::unc-54 3' UTR + LoxP]) IV*
OC1389	*ssna-1(bs382[R18E, R20E, Q98E])/ears-2(ve631[LoxP + myo-2p::GFP::unc-54 3' UTR + rps-27p::neoR::unc-54 3' UTR + LoxP]) IV*

Supplementary Table 3. Repair templates and crRNA sequences used in this study.

Allele	Background	crRNA (5' -> 3')	Repair template (5' -> 3')
bs182	N2	TAGAATCATGCATTTGCATT<br><br>CTTTGTGCGCAAAGAGTATC	TTCGTATTTGAACAATTACTGA<br>CTAATTTCCTCCGAATGCAAA<br>TGCATGATTCTAGAACAAAAA<br>AAACATCAGAAATATTGAACT<br>CTGAACAACTGTCTC
bs218	N2	CTTTGTGCGCAAAGAGTATC<br><br>GATATATTTACAGGCATTTT	GCAAAAGACGTTGGTGGACTT<br>TGTGCGCAAAGAGTATCAAGA<br>TACGAAACATCAGAAATATGA<br>ACCGGAAGCGTGAACTCTGAA<br>CAACTGTCTCCCAAAAATGCC<br>TGTAAATATATCAATTATCGAC<br>ATAACTTC
bs246	N2	CTTTGTGCGCAAAGAGTATG	CAAAAGACGTTGGTGGACTTT<br>GTGCGCAAAGAGTATGAAGAT<br>ACGAAACATCAGAAATATTGA<br>ACTCTGAACAAC
bs284	N2	AAAATGTCTTCTCGATCTAC<br><br>CTGTGAGACGGCGTTCCTCT	TGCATGATTCTAGAACAAAAA<br>ATGTCTTCTCGAAGCACAGGA<br>AGCTTTGATGAAATATCACAG<br>GGTAAGAGCAAATTGAAGATA<br>AACATTTATAGTAATATTTCAG<br>AGATCCAACGTCTCAGAGAGG<br>AACGCCGTCTCACAGAATCGT<br>CGATTCGAAAAA

bs286	N2	CTGTGAGACGGCGTTCCTCT	GAAGATAAACATTTATAGTAATATTTCAGACATCCAAGAGCTCAGAGAGGAACGCCGTCTCACAGAATCGTCGATTCGAAAAATG
bs312	bs284	CTTTGTGCGCAAAGAGTATC	CGCAAAAGACGTTGGTGGACTTTGTGCGCAAAGAGGAGCAGGATACGAAACATCAGAAATATTGAACTCTGAACAAC
bs314	bs286		
bs355	N2		
bs381	N2	AAAATGTCTTCTCGAAGCAC<br>CTGTGAGACGGCGTTCCTCT	TGCATGATTCTAGAACAAAAAATGTCTTCTCGAAGCACAGGAAGCTTTGATGAAATATCACAGGGTAAGAGCAAATTGAAGATAAACATTTATAGTAATATTTCAGAGATCCAAGAGCTCAGAGAGGAACGCCGTCTCACAGAATCGTCGATTCGAAAAA
bs382	bs246	AGAGGAACGCCGTCTCACAG	AGATAAACATTTATAGTAATATTTCAGACATCCAAGAACTCGAAGAGGAACGCCGTCTCACAGAATCGTCGATTCGAAAAA
