# Supplementary material for: Structural insights into SSNA1 self-assembly and its microtubule binding for centriole maintenance
Source: Nat Commun. 2025 Aug 13;16:7512. doi: 10.1038/s41467-025-62696-9 (PMC12350680; doi:10.1038/s41467-025-62696-9)
Supplement: Supplementary file 2 — Description of Additional Supplementary Files [file 41467_2025_62696_MOESM2_ESM.pdf]

Description of Additional Supplementary Files

Supplementary Movie 1. Time-lapse movie of *ssna-1* WT embryos. The embryos expressed GFP::histone, mCherry::b-tubulin, and GFP::SPD-2 (see also Fig. 1D).

Supplementary Movie 2. Time-lapse movie of *ssna-1*(Δ) embryos. The embryos expressed GFP::histone, mCherry::b-tubulin, and GFP::SPD-2 (see also Fig. 1D).
